# Modulation of Oxidative Stress and Hemostasis by Flavonoids from Lentil Aerial Parts

**DOI:** 10.3390/molecules26020497

**Published:** 2021-01-18

**Authors:** Jerzy Żuchowski, Agata Rolnik, Weronika Adach, Anna Stochmal, Beata Olas

**Affiliations:** 1 Department of Biochemistry, Institute of Soil Science and Plant Cultivation State Research Institute, 24-100 Puławy, Poland; jzuchowski@iung.pulawy.pl (J.Ż.); asf@iung.pulawy.pl (A.S.); 2 Department of General Biochemistry, Faculty of Biology and Environmental Protection, University of Łódź, 90-236 Łódź, Poland; agata.rolnik@unilodz.pl (A.R.); weronika.adach@biol.uni.lodz.pl (W.A.)

**Keywords:** lentil leaves, *Lens culinaris*, flavonoids, oxidative stress, hemostasis

## Abstract

While specific metabolites of lentil (*Lens culinaris* L.) seeds and their biological activity have been well described, other organs of this plant have attracted little scientific attention. In recent years, green parts of lentils have been shown to contain diverse acylated flavonoids. This work presents the results of the research on the effect of the crude extract, the phenolic fraction, and seven flavonoids obtained from aerial parts of lentils on oxidative damage induced by H_2_O_2_/Fe to lipid and protein constituents of human plasma. Another goal was to determine their effect on hemostasis parameters of human plasma in vitro. Most of the purified lentil flavonoids had antioxidant and anticoagulant properties. The crude extract and the phenolic fraction of lentil aerial parts showed antioxidant activity, only at the highest tested concentration (50 μg/mL). Our results indicate that aerial parts of lentils may be recommended as a source of bioactive substances.

## 1. Introduction

**Figure 1:**
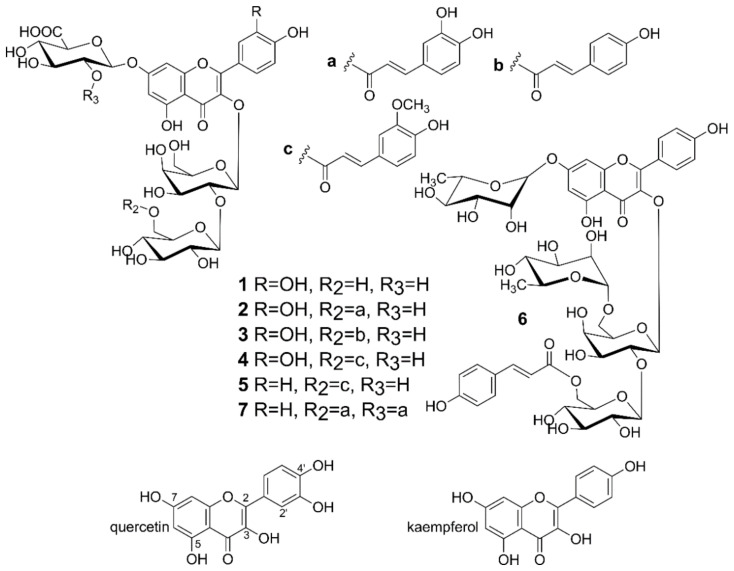
Structures of the investigated flavonoids.

Nowadays, it is widely known that a diet rich in vegetables, fruit and whole-meal cereal products helps in the prevention of age-related diseases, especially cancer, cardiovascular and neurodegenerative diseases. Recently, Tang et al. [1] have described that various vegetables (for example, celery, rape, carrot, lettuce and broccoli) have cardioprotective actions, including modifying lipid metabolism, lowering blood pressure and antioxidant properties. Other vegetables—onion or beetroot are also important diet components for the prevention and treatment of cardiovascular diseases by their anti-aggregatory potential [2]. A broad body of evidence indicates that these health-promoting properties may be attributed to the presence of different plant-specific metabolites, including phenolic compounds [3,4]. For this reason, specific metabolites of crop plants and their biologic activities have been extensively investigated. Lentil (*Lens culinaris* L.) seeds are known as nutrient-rich and healthy food and find also use in traditional medicine in different areas of the world. Lentils were shown to have anticarcinogenic, hypoglycemic, hypocholesterolemic and blood pressure-lowering properties. While phenolic compounds and other specific metabolites of lentil seeds have been very well characterized, little interest has been devoted to the green parts of the plant. However, leaves and stems of lentil were shown to contain diverse acylated glycosides of quercetin and kaempferol [5]. In the current experiment, we focused on the effect of the crude extract, the phenolic fraction and various flavonoids obtained from lentil aerial parts on human plasma—an important element of hemostasis. Plasma oxidative stress may modulate hemostasis and lead to the development of pathological processes of the cardiovascular system [6]. Therefore, the objective was to investigate antioxidant activity of the crude extract, the phenolic fraction and various flavonoids (Figure 1): 4 derivatives of quercetin (compound **1** (quercetin 3-*O*-β-d-glucopyranosyl-(1→2)-β-d-galactopyranoside-7-*O*-β-d-glucuropyranoside); compound **2** (quercetin 3-*O*-[(6-*O*-*E*-caffeoyl)-β-d-glucopyranosyl-(1→2)]-β-d-galactopyranoside-7-*O*-β-d-glucuropyranoside); compound **3** (quercetin 3-*O*-[(6-*O*-*E*-*p*-coumaroyl)-β-d-glucopyranosyl-(1→2)]-β-d-galactopyranoside-7-*O*-β-d-glucuropyranoside); compound **4** (quercetin 3-*O*-[(6-*O*-*E*-feruloyl)-β-d-glucopyranosyl-(1→2)]-β-d-galactopyranoside-7-*O*-β-d-glucuropyranoside)) and 3 derivatives of kaempferol (compound **5** (kaempferol 3-*O*-[(6-*O*-*E*-feruloyl)-β-d-glucopyranosyl-(1→2)]-β-d-galactopyranoside-7-*O*-β-d-glucuropyranoside); compound **6** (kaempferol 3-*O*-{[(6-*O*-*E*-*p*-coumaroyl)-β-d-glucopyranosyl(1→2)]-α-l-rhamnopyranosyl-(1→6)}-β-d-galactopyranoside-7-*O*-α-l-rhamnopyranoside); compound **7** (kaempferol 3-*O*-[(6-*O*-*E*-caffeoyl)-β-d-glucopyranosyl-(1→2)]-β-d-galactopyranoside-7-*O*-(2-*O*-E-caffeoyl’)-β-d-glucuropyranoside)) from lentil aerial parts against the effect of a biological oxidant-H_2_O_2_/Fe (the donor of hydroxyl radicals) on components of human plasma (lipids and proteins). Another aim of our study was also to determine their effect on hemostasis parameters of human plasma in vitro: the activated partial thromboplastin time (APTT), prothrombin time (PT), and thrombin time (TT). The activity of different flavonoids obtained from lentil aerial parts was compared to properties of quercetin and kaempferol.

## 2. Material and Methods

### 2.1. Chemicals

Dimethyl sulfoxide (DMSO), trichloroacetic acid (TCA), thiobarbituric acid (TBA), sodium dodecyl sulfate (SDS), ethylenediaminetetraacetic acid (EDTA), 5,5′-dithiobis-(2-nitrobenzoic acid) (DTNB), formic acid (LC–MS grade), quercetin, kaempferol and H_2_O_2_ were purchased from Sigma (St. Louis, MO, USA). Methanol (isocratic grade) and acetonitrile (LC–MS grade) were acquired from Merck (Darmstadt, Germany). Other reagents represented analytical grade and were provided by commercial suppliers, including POCh, (Gliwice, Poland), Acros (Poznań, Poland), and Chempur (Piekary Śląskie, Poland).

### 2.2. Plant Material

Seeds of lentil (*Lens culinaris* Medik.) cultivar Tina were obtained from the Department of Agrotechnology and Crop Management, University of Warmia and Mazury, Olsztyn, Poland. Lentil plants were grown in the experimental field of the Institute of Soil Science and Plant Cultivation in Puławy, Poland, and harvested during the flowering period. The collected aerial parts of lentil were lyophilized (Gamma 2–16 LSC, Christ, Osterode am Harz, Germany), milled in a laboratory mill, and defatted with chloroform in a Soxhlet extractor (Quickfit, Stone, UK).

### 2.3. Preparation of Extract and Phenolic Fraction from Lentil Aerial Parts

The extract used in this work was prepared according to the earlier described procedure [7]. A 200 g portion of the defatted plant material was extracted with boiling 80% methanol (*v*/*v*; 3 × 2.0 L, for 1 h), under reflux. The obtained extracts were filtered through a sintered glass funnel, rotary evaporated (Heidolph, Schwabach, Germany), and lyophilized. The extraction yield was 35.94 g. The phenolic fraction of the lentil extract was prepared by solid phase extraction (SPE). A 7.03 g portion of the extract was shaken with 1% water–methanol containing 0.1% formic acid, and centrifuged (6654× *g*, 18 °C, 10 min). The supernatant was loaded onto a C18 column (34 × 110 mm; Cosmosil 140C18-Prep, 140 μm). The column was washed with the same solution, and the bound phenolic compounds were subsequently eluted with a 60% methanol solution to yield 976 mg of the phenolic fraction.

### 2.4. Isolation of Flavonoids from Lentil Aerial Parts

Lentil flavonoids were purified from the above-described crude extract by reverse-phase chromatography. The applied isolation procedure included vacuum liquid chromatography, low-pressure liquid chromatography, and semi-preparative HPLC. Structures of the purified compounds were determined by 1D and 2D NMR spectroscopy. A detailed description of isolation and structure elucidation of flavonoids from the aerial parts of lentils can be found in a publication of [7,8].

### 2.5. Stock Solutions

Stock solutions of the crude extract, the phenolic fraction, flavonoids from green parts of lentil (compounds **1**–**7**), quercetin and kaempferol, used in tests of biological activity, were made in 50% DMSO. The final concentration of DMSO in samples was lower than 0.05%, and its effects were determined in all experiments.

### 2.6. Quantification of Flavonoids in the Tested Crude Extract and Phenolic Fraction of the Lentil Aerial Parts

The content of compounds **1**–**7** in the lentil extract and the phenolic fraction was determined by ultra-high-performance liquid chromatographic-photodiode array (UHPLC-PDA) system, using an ACQUITY UPLC chromatographic system equipped with photodiode array (PDA) and triple quadrupole (TQD) mass spectrometer (MS) detectors (Waters Corp., Milford, MA, USA). Samples were separated on an ACQUITY BEH C18 column (2.1 × 100 mm, 1.7 μm; Waters) at 40 °C; the flow rate was 0.400 mL min^−1^, the injection volume was 2.5 μL. The mobile phase was composed of 0.1% (*v*/*v*) formic acid in Milli-Q water (solvent A) and acetonitrile with 0.1% (*v*/*v*) formic acid (solvent B). Elution was performed as follows: 0.0–1.0 min, 5% B; 1.0–15.9 min, 5–27% B; 15.9–16.0 min, 27–99% B; 16.0–17.9 min, 99% B; 17.9–18.0 min, 99–5% B; 18.0–20.5 min, 5% B. Concentration of investigated flavonoids was calculated on the basis of standard curves of the compound **1** (7 points, 2.29–128.10 μg min^−1^; y = 97.63x − 11.83; R^2^ = 0.9999; λ = 255 nm; the standard purity by UHPLC-UV: 86%), compound **2** (8 points, 2.95–236.50 μg min^−1^; y = 178.45x + 60.45; R^2^ = 0.9997; λ = 255 nm; the standard purity by UHPLC-UV: 96%), compound **3** (7 points, 2.83–158.55 μg min^−1^; y = 141.75x − 20.41; R^2^ = 0.9998; λ = 255 nm; the standard purity by UHPLC-UV: 97%), compound **4** (7 points, 3.30–184.80 μg min^−1^; y = 127.59x + 25.67; R^2^ = 0.9997; λ = 255 nm; the standard purity by UHPLC-UV: 96%) and compound **5** (7 points, 1.57–87.85 μg min^−1^; y = 147.57x + 15.38; R^2^ = 0.9999; λ = 265 nm; the standard purity by UHPLC-UV: 87%). The content of compounds **6** and **7** was expressed as the compound 5 equivalent. To better characterize the investigated preparations, four other major flavonoids were additionally determined: 3-*O*-[(6-*O*-*E*-caffeoyl)-β-d-glucopyranosyl-(1→2)]-β-d-galactopyranoside- 7-*O*-β-d-glucuropyranoside (compound **8**), kaempferol 3-*O*-[(6-*O*-*E*-*p*-coumaroyl)-β-d-glucopyranosyl-(1→2)]-β-d-galactopyranoside-7-*O*-β-d-glucuropyranoside (compound **9**), quercetin 3-*O*-[(6-*O*-*E*-caffeoyl)-β-d-glucopyranosyl-(1→2)]-β-d-galactopyranoside-7-*O*-(2-*O*-*E*-caffeoyl’)-β-d-glucuropyranoside (compound **10**), and quercetin 3-*O*-β-d-rhamnopyranoside (compound **11**). Their content was expressed as the compound **1** (quercetin derivatives) or compound **5** (kaempferol derivatives) equivalent.

### 2.7. Human Plasma Isolation

Human blood and plasma were obtained from six regular donors (non-smokers of both sexes) of a blood bank (Lodz, Poland) and a Medical Center (Lodz, Poland). Blood was collected into CPDA solution (citrate/phosphate/dextrose/adenine; 8.5:1; *v*/*v*) or CPD solution (citrate/phosphate/dextrose; 9:1; *v*/*v*). Donors had not taken any medication, alcohol or antioxidant supplementation for a week before donating blood. Analysis of the blood samples was performed according to the guidelines of the Helsinki Declaration for Human Research, with approval of the Committee on the Ethics of Research in Human Experimentation of the University of Lodz (resolution No. 7/KBBN-UŁ/III/2018). For determination of hemostatic parameters, plasma was incubated (30 min, at 37 °C) with:

1. The extract from the lentil aerial parts at the final concentrations of 1–50 μg/mL;
2. The phenolic fraction from lentil aerial parts at the final concentrations of 1–50 μg/mL;
3. Flavonoids (compounds **1**–**7**) from the lentil aerial parts at the final concentrations of 1–50 μg/mL;
4. Quercetin or kaempferol at the final concentrations of 1–50 μg/mL;
5. Human plasma was also pre-incubated (5 min, at 37 °C; for parameters of oxidative stress) with:
6. The extract from the lentil aerial parts at the final concentrations of 1–50 μg/mL;
7. The phenolic fraction from lentil aerial parts at the final concentrations of 1–50 μg/mL
8. Flavonoids (compounds **1**–**7**) from the lentil aerial parts at the final concentrations of 1–50 μg/mL;
9. Quercetin or kaempferol at the final concentrations of 1–50 μg/mL;
10. And then treated with 4.7 mM H_2_O_2_/3.8 mM Fe_2_SO_4_/2.5 mM EDTA (25 min, at 37 °C).

The protein concentration, determined by measuring absorbance at 280 nm (in tested samples), was calculated according to the procedure of Whitaker et al. [9].

### 2.8. Markers of Oxidative Stress

#### 2.8.1. Lipid Peroxidation Measurement

To the test samples after completed incubation, 500 μL of TCA was added, followed by 500 μL of TBA and vortexed for 1 min. Two or three holes were made in Eppendorf caps, which were then heated at 100 °C for 10 min. After incubation, samples were cooled for 15 min at 4 °C and centrifuged at 33,540× *g* at 18 °C for 15 min. The absorbance was measured at 535 nm using the SPECTROstar nano microplate reader (BMG LABTECH, Ortenberg, Germany). The TBARS (thiobarbituric acid reactive substances) concentration was calculated using the molar extinction coefficient (ε = 156,000 M^−1^cm^−1^). More details on the method are described in other papers [10,11].

#### 2.8.2. Carbonyl Group Measurement

Carbonyl content was measured by absorbance at 375 nm (the SPECTROstar nano microplate reader, BMG LABTECH, Ortenberg Germany). The carbonyl group concentration was calculated using a molar extinction coefficient (ε = 22,000 M^−1^ cm^−1^) and the level of carbonyl groups as nmol carbonyl groups/mg of plasma protein. More details on the method are described in other papers [12,13,14].

#### 2.8.3. Thiol Group Determination

After incubation, the test samples were transferred to a 96-well plate at 20 μL, followed by the addition of 20 μL of SDS and mixed thoroughly. Successively, 160 μL of 10 mM phosphate buffer (pH 8.0) was added to all samples and mixed thoroughly. The absorbance was measured at a wavelength λ = 412 nm (A_0_), and 16.6 μL DTNB was added. The 96-well plate was incubated for 60 min (temperature 37 °C). After incubation, the absorbance was measured at a wavelength λ = 412 nm (A_1_). The absorbance difference A_1_-A_0_ was calculated. The thiol group concentration was calculated using the molar extinction coefficient (ε = 13,600 M^−1^ cm^−1^). The level of thiol groups was expressed as μmol thiol groups/mL of plasma. More details on the method are described in other papers [15,16,17].

### 2.9. Parameters of Hemostasis

The prothrombin time (PT), thrombin time (TT) and the activated partial thromboplastin time (APTT) were determined coagulometrically using an optic coagulation analyzer (model K-3002, Kselmed, Grudziadz, Poland), according to the method described by Malinowska et al. [17].

### 2.10. Data Analysis

Several tests were applied to carry out statistical analysis. Six replicates were used in each measurement for this study. In order to eliminate uncertain data, the Dixon Q-test was performed. All the values in this study were expressed as mean ± SD. Obtained results were first analyzed under the account of normality with Shapiro–Wilk test and equality of variance with Levine’s test. Statistical significance of differences among means was assessed by ANOVA (the significance level was *p* < 0.05), followed by Tukey’s multiple comparisons test or Kruskal–Wallis test.

## 3. Results

### 3.1. Quantitative Analysis of the Tested Extract and Phenolic Fraction of the Lentil Aerial Parts

**Figure 2:**
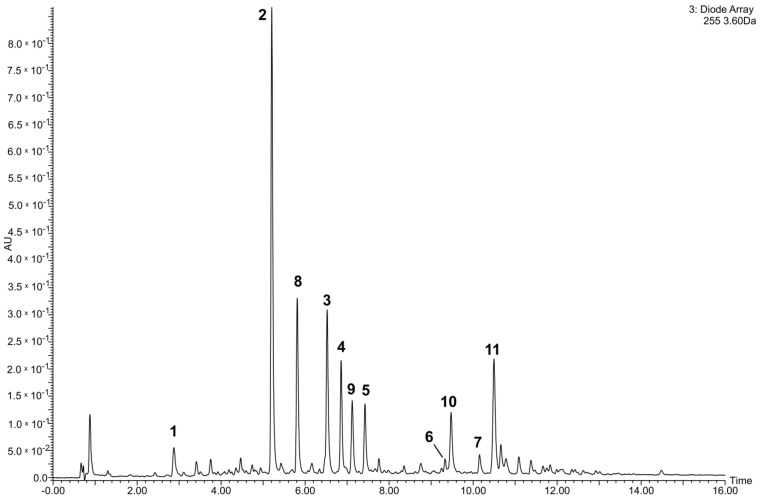
Ultra-high-performance liquid chromatographic-photodiode array chromatogram (λ = 255 nm) of the phenolic fraction of lentil aerial parts (AU—absorbance units). **1**—quercetin 3-*O*-β-d-glucopyranosyl-(1→2)-β-d-galactopyranoside-7-*O*-β-d-glucuropyranoside; **2**—quercetin 3-*O*-[(6-*O*-*E*-caffeoyl)-β-d-glucopyranosyl-(1→2)]-β-d-galactopyranoside-7-*O*-β-d-glucuropyranoside; **3**—quercetin 3-*O*-[(6-*O*-*E*-*p*-coumaroyl)-β-d-glucopyranosyl-(1→2)]-β-d-galactopyranoside-7-*O*-β-d-glucuropyranoside; **4**—quercetin 3-*O*-[(6-*O*-E-feruloyl)-β-d-glucopyranosyl-(1→2)]-β-d-galactopyranoside-7-*O*-β-d-glucuropyranoside; **5**—kaempferol 3-*O*-[(6-*O*-*E*-feruloyl)-β-d-glucopyranosyl-(1→2)]-β-d-galactopyranoside-7-*O*-β-d-glucuropyranoside; **6**—kaempferol 3-*O*-{[(6-*O*-*E*-*p*-coumaroyl)-β-d-glucopyranosyl(1→2)]-α-l-rhamnopyranosyl-(1→6)}-β-d-galactopyranoside-7-*O*-α-l-rhamnopyranoside; **7**—kaempferol 3-*O*-[(6-*O*-*E*-caffeoyl)-β-d-glucopyranosyl-(1→2)]-β-d-galactopyranoside-7-*O*-(2-*O*-*E*-caffeoyl’)-β-d-glucuropyranoside; **8**—kaempferol 3-*O*-[(6-*O*-*E*-caffeoyl)-β-d-glucopyranosyl-(1→2)]-β-d- galactopyranoside-7-*O*-β-d-glucuropyranoside; **9**—kaempferol 3-*O*-[(6-*O*-*E*-*p*-coumaroyl)-β-d-glucopyranosyl-(1→2)]- β-d-galactopyranoside-7-*O*-β-d-glucuropyranoside **10**—quercetin 3-*O*-[(6-*O*-*E*-caffeoyl)-β-d-glucopyranosyl-(1→2)]-β-d-galactopyranoside-7-*O*-(2-*O*-*E*-caffeoyl’)-β-d-glucuropyranoside; **11**—kaempferol 3-*O*-β-d-rhamnopyranoside.

Different glycosides of quercetin and kaempferol, most of them acylated with hydroxycinnamic acids, were the main phenolic constituents of both analyzed lentil preparations. Apart from compounds **1**–**7**, the content of four other flavonoids was additionally evaluated: Compound **2** was the dominant phenolic constituent of both lentil preparations, while **3**–**5** and **8**–**11** were other major phenolics (Table 1, Figure 2). The total content of all determined compounds was 80.8 ± 5.5 mg g^−1^ for the crude extract and 412.5 ± 6.5 mg g^−1^ for the phenolic fraction, respectively.

### 3.2. Effects on Oxidative Stress Biomarkers in Human Plasma *In Vitro*

**Figure 3:**
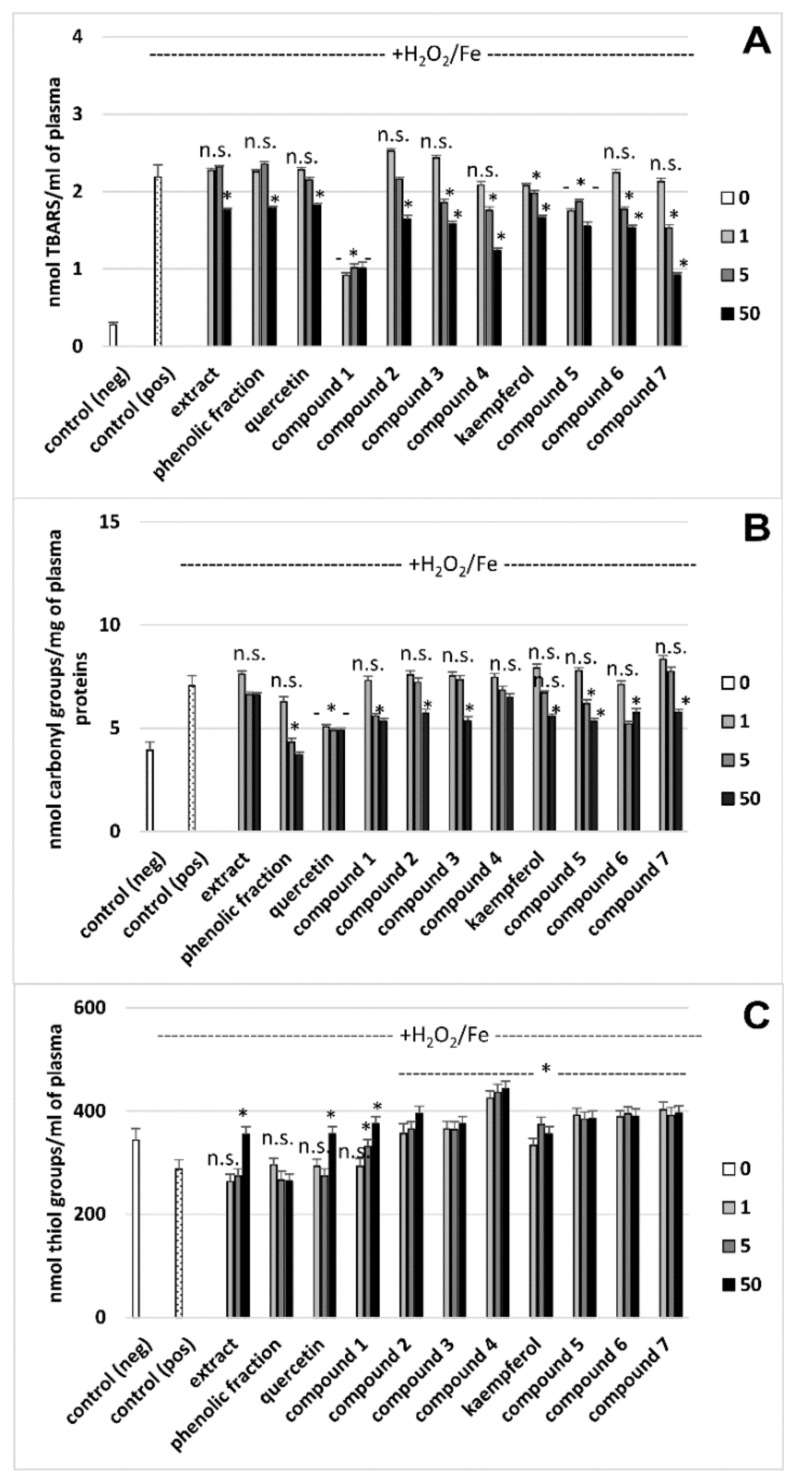
Effects of the crude extract, the phenolic fraction and flavonoids from lentil aerial parts (1–50 μg/mL) and commercial flavonoids (quercetin and kaempferol) on plasma lipid peroxidation (**A**), plasma protein carbonylation (**B**), and oxidation of thiol groups (**C**), induced by H_2_O_2_/Fe. Control negative (neg) refers to plasma not treated with H_2_O_2_/Fe, whereas control positive (pos) to plasma treated with H_2_O_2_/Fe. Data represent means ± SD of 6 independent experiments. * *p* < 0.05 (vs. control (pos)), n.s. (statistically irrelevant) *p* > 0.05 (vs. control (pos)), *p* < 0.05 (between control (neg) and control (pos)); “-“ means the range of concentration of used extracts.

Exposure of plasma to a strong chemical oxidant, H_2_O_2_/Fe, resulted in enhanced plasma lipid peroxidation and plasma protein carbonylation. Compound **1** (at all concentrations) had the strongest antioxidant activity; it reduced the H_2_O_2_/Fe-induced plasma lipid peroxidation by about 60%. This compound was more active than quercetin and its other derivatives (compounds **2**–**4** at 50 μg/mL). Three derivatives of kaempferol (compounds **5**–**7** at 50 μg/mL) were also more active than their aglycone (Figure 3A, Table 2). On the other hand, in this model in vitro, the phenolic fraction (at 5 and 50 μg/mL) caused stronger inhibition of protein carbonylation induced by H_2_O_2_/Fe than tested flavonoids (Figure 3B). The phenolic fraction from the lentil aerial parts (at the highest concentration-50 μg/mL) reduced this process by about 50% (Figure 3B, Table 2). The tested derivatives of quercetin (compounds **1**–**4**), like quercetin, had a similar effect on protein carbonylation induced by H_2_O_2_/Fe. The derivatives of kaempferol also reduced protein carbonylation (about 20% inhibition for 50 μg/mL) (Figure 3B, Table 2).

Analysis of the effect of tested flavonoids, the crude extract and the phenolic fraction from the lentil aerial parts on the level of protein thiols showed that all lentil flavonoids stronger inhibited the thiol H_2_O_2_/Fe-induced oxidation than quercetin and kaempferol (Figure 3C). Quercetin and Compound **1** (at 50 μg/mL) have increased H_2_O_2_/Fe induced oxidation of the thiol protein (Figure 3C). The phenolic fraction was not effective, while the antioxidant activity of the crude extract was observed only at the concentration of 50 μg/mL.

### 3.3. Effects on Hemostatic Parameters of Plasma

**Figure 4:**
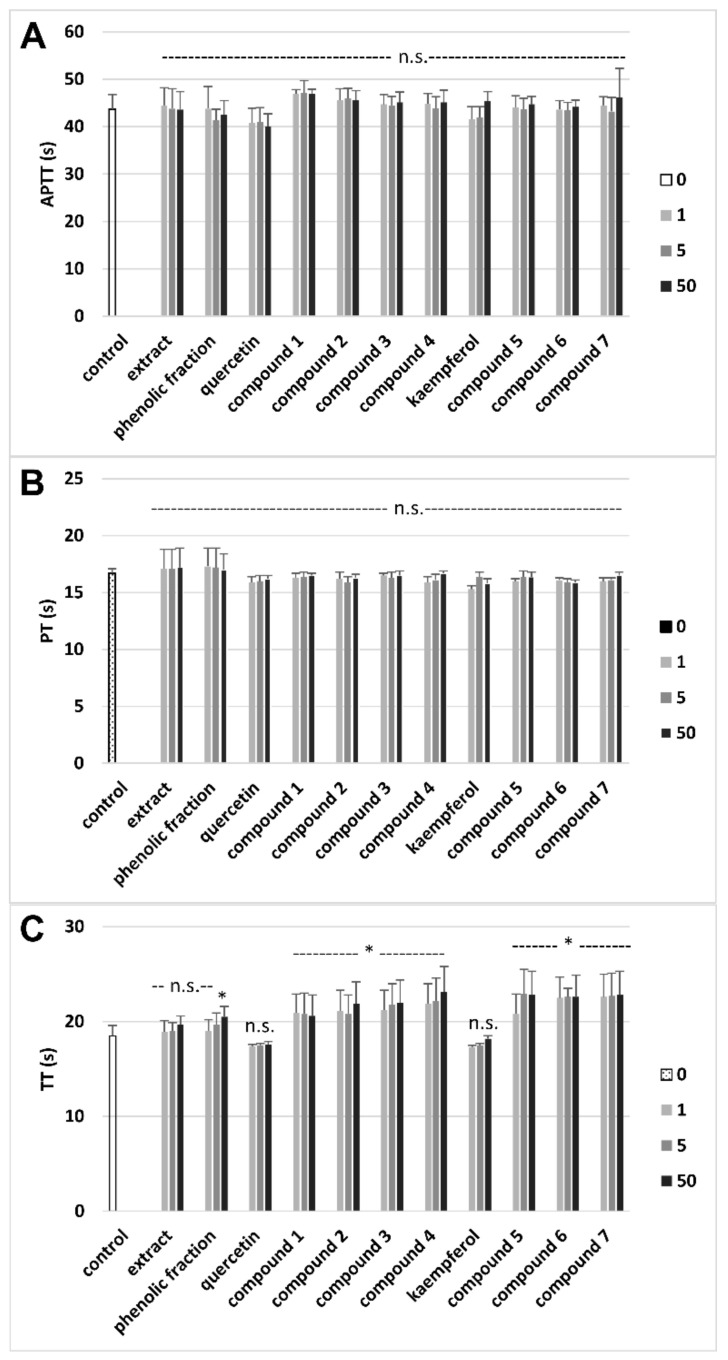
Effects of the crude extract, the phenolic fraction and flavonoids from lentil aerial parts (1–50 μg/mL) and commercial flavonoids (quercetin and kaempferol) on selected hemostatic parameters of plasma: the activated partial thromboplastin time (APTT) (**A**), the prothrombin time (PT) (**B**) and the thrombin time (TT) (**C**). Data represent means ± SD of 6 independent experiments. * *p* < 0.05 (vs. control), n.s. *p* > 0.05 (vs. control).

Analysis of the influence of the tested preparations on the coagulation properties of plasma showed that four derivatives of quercetin-compounds **1**–**4**, and three derivatives of kaempferol-compounds **5**–**7**, significantly prolonged the TT, at the whole tested range 1–50 μg/mL (Figure 4C). However, none of the investigated flavonoids tested crude extract, and the phenolic fraction from lentil aerial parts changed the APTT and PT (Figure 4A,B, respectively). In addition, Table 2 demonstrates comparative effects of the crude extract, the phenolic fraction and flavonoids from lentil aerial parts and commercial two flavonoids (at the highest used concentration-50 μg/mL) on the TT.

## 4. Discussion

The presence of phenolic compounds in fruits and vegetables is correlated with the beneficial effects of these food products on human health. For example, various biological actions of fruits (including berries) against diseases associated with oxidative stress have been attributed to their high phenolic antioxidant content, especially phenolic acids [2]. It has known that phenolic compounds are effective agents preventing damages related to oxidative stress, which plays a crucial role in the etiology and progression of various diseases, for example, cardiovascular diseases [1]. Besides, it has been demonstrated that flavonoids, including kaempferol and quercetin, have cardioprotective action [18]. These compounds consist of two phenyl rings: A and B, connected to a heterocyclic ring-ring C. The chemical activity of these compounds depends on the number of hydroxyl groups [19,20]. Choi et al. [21] demonstrated the cardioprotective effect of kaempferol in in vivo and ex vivo experiments. Two animal models were used in studies, male mice (Imprinting Control Region, outbred strain) and male rats (Sprague–Dawley). Results in in vivo model show that kaempferol protected against thrombosis development in thrombin- and collagen/epinephrine-induced acute thromboembolism models and a FeCl_3_-induced carotid arterial thrombus model. The anticoagulant effect was further confirmed in an ex vivo experiment in mice models [21].

The antioxidant properties of lentil seed extracts are well documented. Their antioxidant activity was usually determined using DPPH (2,2-diphenyl-1-picrylhydrazyl radical), ABTS^⦁^, TEAC, FRAP (fluorescence recovery after photobleaching), and/or ORAC assays, but their influence on lipid peroxidation processes was also occasionally reported [22,23,24,25,26,27]. The antiradical activity of different flavonoids purified from the aerial parts of the lentil was determined using DPPH^⦁^ method [7]. Moreover, our earlier results indicate that quercetin and kaempferol derivatives isolated from aerial parts of the lentil modulate blood platelet function [8]. In the present work, for the first time, we characterized the influence of the crude extract and phenolic fraction from the aerial parts of lentils, as well as their constituent flavonoids on H_2_O_2_/Fe-induced lipid peroxidation, protein carbonylation, and oxidation of protein thiols in human plasma in vitro.

Quercetin and kaempferol are among the most ubiquitous flavonoid aglycones. Our current and earlier report showed that their glycosides were the main phenolics of lentil aerial parts [7]. Quercetin glycosides constituted about 73% of the total determined flavonoids. Among them, compounds **2**–**4** were dominant, constituting about 46% of the total flavonoids (Table 1). For this reason, these three flavonoids may be expected to have had the strongest influence on the biological activity of the crude extract and phenolic fraction of lentil. Similarly, the influence of compounds **1**, **6**, and **7** on the bioactivity of these preparations was most probably negligible due to their low content.

The majority of flavonol glycosides from lentil leaves and stems seem to be unique, not reported from any other plant. They share a common glycosylation pattern, and most of them are acylated with hydroxycinnamic acids (e.g., compounds **2**–**5**, and **7**–**10**). Moreover, they are 7-*O*-glucuronides, and this kind of flavonol glycosides apparently occurs rarely in plants. Phenolic compound profiles of lentil seeds and lentil aerial parts were completely different. The extract from the seeds of lentil cv. Tina contained significant amounts of a non-acylated flavonol glycoside (a kaempferol dihexoside-dideoxyhexoside) but seemed to be devoid of flavonol glucuronides, which were dominant in the extract from lentil leaves and stems [7]. The literature data indicate the presence of proanthocyanidins, catechin and epicatechin, phenolic acids and flavonol glycosides (but not flavonol glucuronides) in lentil seeds [23,24]. We did not determine the content of phenolic compounds in the extract from seeds of lentil cv. Tina; however, the total content of all determining phenolic compounds in the 80% acetone extract from seeds of green lentil (~1 mg g^−1^) was about 80 times lower than that in the currently characterized extract from the aerial parts of lentil [24].

The lentil extract and phenolic fraction inhibited plasma lipid peroxidation only at the highest tested concentration. Pure flavonol glycosides (compounds **1**–**7**) showed distinctly stronger antioxidant activity (though usually not at the lowest applied concentration), while flavonoid aglycons turned out to be surprisingly weak inhibitors of lipid peroxidation, especially quercetin, the activity of which was comparable to those of the crude extract and phenolic fraction. It can be observed, especially at the highest tested concentration, that inhibitory activity of acylated flavonoids tended to increase with their hydrophobicity (compounds **2**–**4** and **5**–**7**), which can be explained by better interactions of fewer polar compounds with plasma lipids. This pattern is disturbed by compound **1**, a non-acylated quercetin glycoside, showing the strongest inhibitory properties of all investigated compounds. This observation, as well as the low antioxidant activities of quercetin and kaempferol, seem to be not easy to explain and may be partly attributed to the applied experimental conditions. Flavonoids are known as efficient free radical scavengers and chelator of heavy metal ions. Flavonoid aglycones are commonly regarded as more efficient radical scavengers than their glycosides. Possibly, the chelation of Fe^2+^ ions strongly influenced the observed antioxidant activity in our experiments and compounds **1**–**7** were generally more efficient Fe^2+^ chelators than their aglycones. Similarly, rutin (quercetin 3-*O*-rutinoside) was found to be a stronger inhibitor of Fe^2+^-induced linoleate peroxidation than quercetin, which was attributed to the ability of rutin to form inert complexes with iron [28]. There is no available data concerning the biological activity of lentil leaf and stem flavonoids. Comparable lipid peroxidation experiments using blood plasma and H_2_O_2_/Fe—induced oxidative stress are also not common. However, our earlier work on sea buckthorn fruit flavonoids also demonstrated that two isorhamnetin glycosides exerted a similar and stronger inhibitory effect on plasma lipid peroxidation as compared to their aglycone [14].

As regards other antioxidant experiments, acylated flavonoids and kaempferol provided the highest protection of thiol groups, at all concentrations, among all tested compounds and preparations; the phenolic fraction was not active, while the protective effect of the crude extract and quercetin was observed only at the highest dose. The antioxidant activity of kaempferol and its derivates by decreasing the production of reactive oxygen species due to inhibition of pro-oxidant enzymes and activation of antioxidant enzymes. Kaempferol and derivates are also the patent scavengers of superoxide anion and hydroxyl radical [29]. Except for the crude extract and the compound **4**, the investigated substances also significantly reduced the carbonylation of plasma proteins, at least at the highest dose; quercetin and the phenolic fraction had the highest inhibitory activity. The antioxidant mechanism of quercetin is based on directed scavenging of reactive oxygen species, chelation of metals involved in the generation of reactive oxygen species and inhibition of enzymes generating reactive oxygen species [30]. Zhou et al. [31] demonstrated the antioxidant activity of kaempferol in in vivo studies on a rat model. The animals were divided into four groups: the control group, the ischemia-reperfusion injury group, the kaempferol group and the 4-benzyl-2-methyl-1,2,4-thiadiazolidine-3,5-dione group. Oxidative stress was analyzed by measuring the level of superoxide dismutase (SOD), malondialdehyde (MDA) and glutathione disulfide ratio. The obtained results demonstrated the antioxidant activity of kaempferol due to the increased level of SOD and decreased MDA level in the kaempferol group compared to the control group [31].

Oxidative stress is very often linked with modulation of hemostasis and cardiovascular disorders. The coagulation process (also known as clotting) is an important element of hemostasis, and it includes blood changes from a liquid to a gel, forming a blood clot. In our experiment, we measured different coagulation times (TT, PT, and APTT) using a coagulometer. One of the key findings of our experiments is a demonstration of anticoagulant properties of derivatives of quercetin and kaempferol isolated from lentil aerial parts. These compounds prolonged clotting time—the TT of human plasma. We suppose that the anticoagulant activity of tested flavonoids may be associated with a modulation of thrombin activity. Similar effects have been observed in other experiments. The results of Choi et al. [32] indicate that various flavonoids, including quercetin 3-*O*-β-d-glucoside, may inhibit the enzymatic activity of thrombin [32]. Liu et al. [33] also proved that flavonoids could inhibit the enzymatic activity of thrombin. The effect of various natural flavonoids, including quercetin and kaempferol, on thrombotic time, was tested. Both kaempferol and quercetin extend the thrombotic time [33].

The wide range of applied concentrations of the crude extract, the phenolic fraction, and phenolic compounds isolated from lentil aerial parts (1–50 μg/mL) was in accordance with the general practice in in vitro model [11,14,17]. Besides, the concentration range used in studies was the same as in our earlier studies to maintain the continuity of research [8]. Moreover, the lower concentrations (1 and 5 μg/mL) may be considered as physiologically achievable after consumption of phenolic–rich plant materials [34]. For example, the maximal achievable concentration of plant phenolic compounds in plasma can reach up to 5 μg/mL [34,35]. However, foods with a high concentration of kaempferol and quercetin are not necessarily the most bioavailable source. The absorbed these flavonoids are metabolized in the liver and circulate as glucuronide, methyl, and sulfate metabolites [18]. On the other hand, recently, Stainer et al. [36] have observed that two quercetin metabolites–isorhamnetin and tamarixetin possess antithrombotic properties. For example, these compounds inhibited blood platelet activation, including platelet aggregation, granule secretion, calcium mobilization, and integrin α_IIb_β_3_ function. In addition, isorhamnetin had antioxidant and anticoagulant activity [14].

In conclusion, the present paper is the first detailed study on the biological activity of the extract, the phenolic fraction, and pure phenolic compounds from the aerial parts of lentils. We also observe that not only tested extract and fraction but also quercetin and kaempferol derivatives have antioxidant and sometimes additionally anticoagulant potential. The results reveal that lentil aerial parts may be recommended as a material for use in functional food products. However, though antioxidant and anticoagulant properties of the investigated preparations were demonstrated in vitro in human plasma, their real effect should be verified in in vivo models.

## Figures and Tables

**Table 1:** Content of the compounds **1**–**7** and other major flavonol glycosides (mg g^−1^; mean value ± SD) in the crude extract (CE) and the phenolic fraction (PF) of lentil aerial parts.

Compound	Preparations	
	CE	PF
**1**	3.4 ± 0.2	16.5 ± 0.1
**2**	20.5 ± 1.3	103.4 ± 1.7
**3**	10.1 ± 0.8	50.7 ± 0.8
**4**	6.9 ± 0.5	35.2 ± 1.2
**5**	4.2 ± 0.2	22.0 ± 0.4
**6**	1.0 ± 0.1 ^a^	5.6 ± 0.1 ^a^
**7**	1.1 ± 0.1 ^a^	5.5 ± 0.3 ^a^
**8**	9.6 ± 0.6 ^a^	48.8 ± 0.9 ^a^
**9**	5.5 ± 0.4 ^a^	28.2 ± 0.5 ^a^
**10**	6.4 ± 0.4 ^b^	33.3 ± 1.0 ^b^
**11**	12.2 ± 0.8 ^b^	63.2 ± 1.0 ^b^

^a^—equivalent of the compound **5**; ^b^—equivalent of the compound **1**.

**Table 2:** Comparative effects of the crude extract, the phenolic fraction and flavonoids from lentil aerial parts and commercial flavonoids (quercetin and kaempferol) (50 μg/mL) on selected parameters of oxidative stress and hemostasis. Data represent means ± SD of 6 independent experiments.

Tested Extract/Fraction/Phenolic Compound	Parameters of Oxidative Stress			Parameters of Hemostasis
	Inhibition of Lipid Peroxidation Induced by H_2_O_2_/Fe (%)	Inhibition of Protein Carbonylation Induced by H_2_O_2_/Fe (%)	The Level of Protein Thiol Groups (nmol/mL of Plasma) in Plasma Treated with H_2_O_2_/Fe	Prolongation of TT (the Thrombin Time) (%)
Extract (a)	19.4 ± 4.8 Antioxidant action vs. control (plasma treated with H_2_O_2_/Fe)	6.2 ± 1.9 (*p* > 0.05) Antioxidant action vs. control (plasma treated with H_2_O_2_/Fe)	355.2 ± 13.7 Antioxidant action vs. control (plasma treated with H_2_O_2_/Fe)	No effect vs. control
Phenolic fraction (b)	18.2 ± 3.8 (*p* > 0.05; b vs. a) Antioxidant action vs. control (plasma treated with H_2_O_2_/Fe)	47.3 ± 7.9 (*p* < 0.05; b vs. a) Antioxidant action vs. control (plasma treated with H_2_O_2_/Fe)	263.8 ± 15.2 (*p* < 0.05; b vs. a) No effect vs. control (plasma treated with H_2_O_2_/Fe)	10.8 ± 2.4 Anticoagulant action vs. control
Quercetin (c)	16.5 ± 3.5 (*p* > 0.05; c vs. a, b)Antioxidant action vs. control (plasma treated with H_2_O_2_/Fe)	30.4 ± 10.9 (*p* < 0.05; c vs. a; *p* > 0.05; c vs. b) Antioxidant action vs. control (plasma treated with H_2_O_2_/Fe)	355.7 ± 13.7 (*p* > 0.05; c vs. a; *p* < 0.05; c vs. b) Antioxidant action vs. control (plasma treated with H_2_O_2_/Fe)	No effect vs. control
Compound **1** (d)	53.8 ± 12.7 (p < 0.05; d vs. a, b, c, e, f; *p* > 0.05; d vs. g) Antioxidant action vs. control (plasma treated with H_2_O_2_/Fe)	21.4 ± 8.7 (*p* > 0.05; d vs. c; *p* < 0.05; d vs. a, b) Antioxidant action vs. control (plasma treated with H_2_O_2_/Fe)	375.1 ± 11.6 ( *p* > 0.05; d vs. a, c; *p* < 0.05; d vs. b) Antioxidant action vs. control (plasma treated with H_2_O_2_/Fe)	11.1 ± 2.90 (*p* < 0.05; d vs. c) Anticoagulant action vs. control
Compound **2** (e)	25.0 ± 6.9 (*p* < 0.05; e vs. c; *p* > 0.05; e vs. a, b) Antioxidant action vs. control (plasma treated with H_2_O_2_/Fe)	18.5 ± 6.0 (*p* > 0.05; e vs. c; *p* < 0.05; e vs. a, b) Antioxidant action vs. control (plasma treated with H_2_O_2_/Fe)	395.1 ± 20.2 (*p* > 0.05; e vs. a, c; *p* < 0.05; e vs. b) Antioxidant action vs. control (plasma treated with H_2_O_2_/Fe)	18.3 ± 3.6 (*p* < 0.05; e vs. c) Anticoagulant action vs. control
Compound **3** (f)	27.7 ± 7.8 (*p* < 0.05; f vs. c; (*p* > 0.05; f vs. a, b) Antioxidant action vs. control (plasma treated with H_2_O_2_/Fe)	24.0 ± 7.0 (*p* > 0.05; f vs. c; *p* < 0.05; f vs. a, b) Antioxidant action vs. control (plasma treated with H_2_O_2_/Fe)	375.4 ± 16.4 (*p* > 0.05; f vs. a, c; *p* < 0.05; f vs. b) Antioxidant action vs. control (plasma treated with H_2_O_2_/Fe)	18.9 ± 5.4 (*p* < 0.05; f vs. c) Anticoagulant action vs. control
Compound **4** (g)	43.3 ± 10.5 (*p* < 0.05; g vs. a, b, c) Antioxidant action vs. control (plasma treated with H_2_O_2_/Fe)	7.8 ± 10.5 (*p* > 0.05; g vs. a, c; *p* < 0.05; g vs. b) Antioxidant action vs. control (plasma treated with H_2_O_2_/Fe)	443.6 ± 17.6 (*p* < 0.05; g vs. a, b, c. d, f) Antioxidant action vs. control (plasma treated with H_2_O_2_/Fe)	24.9 ± 6.8 (*p* < 0.05; g vs. b, c, d) Anticoagulant action vs. control
Kaempferol (h)	23.9 ± 4.9 (*p* > 0.05; h vs. a, b) Antioxidant action vs. control (plasma treated with H_2_O_2_/Fe)	20.8 ± 3.9 (*p* < 0.05; h vs. a, b) Antioxidant action vs. control (plasma treated with H_2_O_2_/Fe)	356.7 ± 14.2 (*p* > 0.05; h vs. a; *p* < 0.05; h vs. b) Antioxidant action vs. control (plasma treated with H_2_O_2_/Fe)	No effect vs. control
Compound **5** (i)	29.0 ± 5.0 (*p* > 0.05; i vs. a, b, h) Antioxidant action vs. control (plasma treated with H_2_O_2_/Fe)	24.5 ± 4.8 (*p* > 0.05; i vs. h; *p* < 0.05; i vs. a, b) Antioxidant action vs. control (plasma treated with H_2_O_2_/Fe)	358.8 ± 14.8 (*p* > 0.05; i vs. a, h; *p* < 0.05; i vs. b) Antioxidant action vs. control (plasma treated with H_2_O_2_/Fe)	23.3 ± 6.7 (*p* < 0.05; i vs. b, h) Anticoagulant action vs. control
Compound **6** (j)	29.8 ± 4.9 (*p* < 0.05; j vs. h; *p* > 0.05; j vs. a, b) Antioxidant action vs. control (plasma treated with H_2_O_2_/Fe)	18.2 ± 3.7 (*p* > 0.05; j vs. h; *p* < 0.05; j vs. a, b) Antioxidant action vs. control (plasma treated with H_2_O_2_/Fe)	390.3 ±14.2 (*p* > 0.05; j vs. a, h; *p* < 0.05; j vs. b) Antioxidant action vs. control (plasma treated with H_2_O_2_/Fe)	22.2 ± 5.9 (*p* < 0.05; j vs. b, h) Anticoagulant action vs. control
Compound **7** (k)	57.8 ± 14.9 (*p* < 0.05; k vs. a, b, h, i, j) Antioxidant action vs. control (plasma treated with H_2_O_2_/Fe)	18.1 ± 3.9 (*p* > 0.05; k vs. h; *p* < 0.05; k vs. a, b) Antioxidant action vs. control (plasma treated with H_2_O_2_/Fe)	396.1 ± 14.3 (*p* > 0.05; k vs. a, b, h) Antioxidant action vs. control (plasma treated with H_2_O_2_/Fe)	23.2 ± 7.2 (*p* < 0.05; k vs. b, h) Anticoagulant action vs. control

## Data Availability

Samples of plant extract and phenolic compounds are available from the authors.

## Sample Availability

Samples of the compounds are available from the authors.

## Abbreviations

TBARS	thiobarbituric acid reactive substances
APTT	the activated partial thromboplastin time
PT	the prothrombin time
TT	the thrombin time
n.s.	statistically irrelevant
